# Simplifying Thromboprophylaxis Could Improve Outcomes in Orthopaedic Surgery

**DOI:** 10.1155/2010/108049

**Published:** 2010-09-13

**Authors:** Richard J. Friedman

**Affiliations:** _1_ Department of Orthopaedic Surgery, Roper Hospital, Charleston Orthopaedic Associates, 1012 Physicians Drive, Charleston, SC 29414, USA

## Abstract

Venous thromboembolism is a serious complication after total hip or knee surgery, and there is a well-established clinical need for thromboprophylaxis. However, in a large number of cases adequate administration of thromboprophylaxis does not seem to occur after total joint arthroplasty. A major challenge in the management of thromboprophylaxis is to balance the benefits of treatment with the risks, including bleeding complications. Another potential barrier to the optimal use of thromboprophylaxis could be the inconvenience of currently available agents. Many surgeons therefore adopt a conservative approach towards thromboprophylaxis. Simplifying therapy with more convenient, efficacious, and safe anticoagulants could change attitudes to anticoagulant use and improve adherence to thromboprophylactic guidelines.

## 1. Introduction

Venous thromboembolism (VTE) is a serious complication after major orthopaedic surgery [1]. The rates of venographic deep vein thrombosis (DVT) and proximal DVT 7 to 14 days after major orthopaedic surgery in patients who receive no thromboprophylaxis are approximately 40% to 60% and 10% to 30%, respectively [1]. The manifestation of DVT is, to some extent, a consequence of bone damage during surgery, when procoagulant debris triggers thrombin generation, resulting in hypercoagulability [2]. In addition to hypercoagulability, the other components of Virchow's triad of venous stasis and endothelial damage are also thought to play a part in thrombosis [3]. Thus, there is a well-established clinical need for thromboprophylaxis after arthroplasty [1].

A major challenge in the management of anticoagulants is to balance the benefits of treatment with the risks, including bleeding complications. Many surgeons appear concerned about postoperative bleeding and tend to adopt a conservative approach towards the relative risks and benefits of thromboprophylaxis [2]. Consequently, although evidence-based guidelines and recommendations advocate the use of anticoagulants after major orthopaedic surgery, thromboprophylaxis is still used suboptimally [4–6]. However, the evidence that careful prophylaxis administered at an appropriate time after surgery causes surgical bleeding is sparse [7]. In this paper, current trends in thromboprophylaxis after orthopaedic surgery in the United States (US) are described. Factors limiting appropriate implementation of thromboprophylaxis regimens are also discussed.

## 2. Current Standard of Care

Further to the consensus document developed by the National Institute of Health in 1986 [8], there have been a series of American College of Chest Physicians (ACCP) guidelines published on the use of pharmacological agents for thromboprophylaxis after total hip arthroplasty (THA) and total knee arthroplasty (TKA), last updated in 2008 [1].

In the US, the available options for anticoagulation and thromboprophylaxis after elective THA or TKA are the vitamin K antagonists (VKAs, e.g., warfarin), the low-molecular-weight heparins (LMWHs), and fondaparinux (an indirect Factor Xa inhibitor). Each of these options is associated with significant limitations that complicate use in clinical practice. VKAs have been the mainstay of oral anticoagulant therapy for more than 60 years [9]. However, VKAs have unpredictable pharmacokinetics and pharmacodynamics and significant inter- and intrapatient variability in dose-response relationships. They are associated with multiple drug-drug and food-drug interactions and have a narrow therapeutic window [9]. Regular coagulation monitoring is therefore required to ensure that the international normalized ratio is within the recommended range of 2.0 to 3.0. The heparins are administered subcutaneously, which means that patients often require daily appointments or a nurse visit to administer their medication. LMWHs are also associated with the risk of developing heparin-induced thrombocytopenia [10]. Fondaparinux is also administered subcutaneously and is contraindicated in patients with severe renal impairment and in those that weigh less that 50 kg. In patients over the age of 75 who have undergone THA or TKA, fondaparinux causes an increased risk of bleeding [11].

The timing of initiation of prophylaxis depends upon the type of anticoagulant used. Warfarin therapy is generally initiated prior to surgery because of its delayed onset of action whereas prophylaxis with LMWH can be started 10–12 hours before or 12–24 hours after surgery. There does not seem to be a clear advantage with either regimen, and both regimens are recommended by the ACCP [1]. Thromboprophylaxis is recommended to continue for at least 10 days after joint replacement surgery, with extended prophylaxis for up to 35 days recommended for those patients undergoing THA surgery and with a suggestion that thromboprophylaxis for up to 35 days could be beneficial for those undergoing TKA [1]. Traditionally, thromboprophylaxis used to continue only until the patient was discharged from hospital [12] despite the fact that this could be a suboptimal duration [13] and the risk of DVT and mortality after discharge is considerable [14, 15]. The median length of stay in US hospitals is now as short as 3 days after THA and 4 days after TKA [16]. A retrospective study of the medical records of 3,778 orthopaedic surgery patients found that 88% were discharged from hospital and prescribed warfarin or acetylsalicylic acid [6].

## 3. Suboptimal Utilization of Thromboprophylaxis

Despite the fact that thromboprophylaxis is now recommended for routine use after total joint arthroplasty, it is not always used optimally. Approximately 10% of patients received inadequate in-hospital thromboprophylaxis, and approximately 33% received inadequate postdischarge thromboprophylaxis according to findings from the US Hip and Knee Registry (1996–2001) [17]. An analysis of the data from the multinational Global Orthopaedic Registry (GLORY) to evaluate the compliance of surgeons with the ACCP guidelines for the prevention of VTE showed that only 47% of THA patients and 61% of TKA patients received prophylaxis in accordance with the recommended start time, duration, and dose/treatment intensity recommended by the guidelines [16]. Although nearly all patients received prophylaxis on the first day after surgery, more than a quarter did not receive any form of prophylaxis 7 days after surgery [18]. 

Suboptimal thromboprophylaxis decreases patient outcomes, resulting in many patients remaining at unnecessary risk of thrombosis and its complications [4]. The reasons for lack of compliance with the guidelines may be numerous. They include lack of awareness, poor understanding or disagreement with guidelines (either specifically or as a general concept), resistance to changing established practices, and doubt that a new approach will change outcomes. Established surgeons may also be reluctant to use new anticoagulant regimens because of a fear of increased bleeding risk [17]. Attitudes may also limit a physician's willingness to follow guidelines. An awareness of the guidelines does not necessarily mean that physicians have sufficient knowledge to critically evaluate and apply recommendations [4]. 

Other potential barriers include the mistaken belief that a small asymptomatic DVT is not important because it cannot cause clinically significant pulmonary embolism (PE) [19], which fortunately is only held by a minority [20]. Due to the often clinically silent nature of VTE, and the low incidence of VTE during the short postoperative hospital stay, the chances of a surgeon witnessing a major DVT or an acute PE are rare [4]. In addition, the trend towards earlier hospital discharge means that many symptomatic events occur after that time [21, 22], and patients are often seen by other specialists when referred back to hospital with a venous thromboembolic event; therefore, surgeons are often unaware of the true incidence of VTE in their patients. 

Long-term sequelae of VTE are frequent and often disabling [23]. Recurrent VTE can occur after surgery although the incidence is less than in other patients groups such as those with cancer [24]. Thrombosis damages the deep venous valves resulting in venous reflux and venous hypertension of the lower limbs. This residual venous obstruction and inflammation are thought to be responsible for the development of postthrombotic syndrome [25, 26]. Chronic thrombotic pulmonary hypertension, which is associated with considerable morbidity and mortality, occurs in approximately 3%-4% of patients over 2 years after a symptomatic PE [27].

## 4. Economic Impact of Venous Thromboembolism

The acute and chronic phases of VTE-related care have substantial economic consequences [28, 29] that can be effectively modeled [30]. A recent study found that the total annual healthcare cost for a VTE ranged from $7,594 to $16,644, depending on the type of event and whether it was a primary or secondary diagnosis. The hospital readmission rates for DVT or PE within 12 months were 5.3% for primary and 14.3% for secondary diagnoses [31]. These data indicate that thromboprophylaxis with anticoagulants should not only be beneficial to patients but could also be cost effective for the healthcare system [32, 33].

## 5. Need for More Convenient Anticoagulants

Another potential barrier to the optimal use of thromboprophylaxis could be the inconvenience of currently available agents [34]. Orthopaedic surgeons and their patients would benefit from an oral anticoagulant that could be administered in fixed doses [35].

## 6. Simplifying Therapy

Noncompliance can result in a poor quality of life and increased medical expenditures in managed care. In a study of diabetic patients, total medical costs were approximately $4,500 for patients at 80%–100% adherence compared with approximately $8,900 for those at 1%–19% adherence [36]. A variety of factors affect noncompliance, but simplifying treatment has been shown to improve adherence in asthma patients [37], and cardiovascular patients given single-pill amlodipine/atorvastatin were found to be approximately three times more likely to achieve adherence over 1 year of followup than patients given a two-pill regimen [38]. Similarly, simplifying therapy to a once-daily regimen in virologically suppressed HIV-1-infected patients improved adherence and patient satisfaction [39].

## 7. Novel Anticoagulants

Anticoagulants in development are targeting different steps in the coagulation pathway to provide simpler alternatives to currently available anticoagulants. Among these new agents are direct thrombin inhibitors and direct Factor Xa inhibitors [40]. The direct thrombin inhibitor dabigatran etexilate appears an attractive alternative to the current standard of care in patients after THA and TKA [41–44]. It has been granted marketing authorization in the European Union and Canada for the prevention of VTE after THA or TKA. The manufacturer's recommended dose is 220 mg once daily (starting 1–4 hours after surgery with a single 110 mg capsule) for a total of 28–35 days after THA or a total of 10 days after TKA [45]. Direct Factor Xa inhibitors in development include rivaroxaban, apixaban, edoxaban (DU-176b), and YM150, and of these rivaroxaban is in the most advanced stage of development [46]. Rivaroxaban has shown potential as a once-daily, oral anticoagulant that may be administered in fixed doses for the prevention and treatment of thromboembolic disorders following orthopaedic surgery [47–52]. Rivaroxaban is approved in more than 90 countries worldwide, including the European Union and Canada, for the prevention of VTE after elective hip or knee replacement surgery in adult patients. A dose of 10 mg once daily (with the initial dose 6–10 hours after surgery, provided that haemostasis has been achieved) for 5 weeks after elective hip arthroplasty or 2 weeks after elective knee arthroplasty is recommended by the manufacturer [53].

The main difference between direct thrombin inhibitors and direct Factor Xa inhibitors is their mechanism of action. They also differ in their pharmacokinetic and pharmacodynamic profiles, such as metabolism. For example, in the case of dabigatran, more than 80% of the systemically available drug is eliminated by renal excretion [54]. Two-thirds of administered rivaroxaban are metabolized to inactive metabolites (half of this is eliminated via the kidneys and half via the fecal route), and one-third is excreted as unchanged active drug in the urine [55].

## 8. Conclusion

The need to use thromboprophylaxis after major orthopaedic surgery is now becoming well recognized. However, adequate administration of thromboprophylaxis regimens does not seem to occur after total joint arthroplasty in a large number of cases. The reasons for this appear complex, involving surgeons' poor awareness of the problem of post-surgical thrombosis, their attitudes to guidelines, concerns about causing bleeding, and the complexities of anticoagulation with current agents. Simplifying therapy, such as once-daily fixed dosing, could change attitudes to anticoagulant use and improve adherence to guidelines. Newly developed, oral, fixed-dose anticoagulants should enable substantial improvement in thromboprophylaxis usage, thereby improving patient outcomes. The primary drawback of the new anticoagulants, particularly those with a long half-life, is the lack of specific antidotes to reverse their anticoagulant effect [56]. Specific antidotes might be needed in particular situations such as for overdose or emergency surgery. However, this may not pertain to dabigatran and rivaroxaban as they have relatively short half-lives (12–14 hours and 7–11 hours, resp.) [45, 53]. As off-label prescribing is not uncommon, there is a risk that new anticoagulants licensed for thromboprophylaxis after THA or TKA will be prescribed for unlicensed indications [57]. These current challenges could be overcome by finding specific antidotes and postapproval surveillance of off-label prescribing.
